# Cardiorespiratory Monitoring during Neonatal Resuscitation for Direct Feedback and Audit

**DOI:** 10.3389/fped.2016.00038

**Published:** 2016-04-18

**Authors:** Jeroen J. van Vonderen, Henriëtte A. van Zanten, Kim Schilleman, Stuart B. Hooper, Marcus J. Kitchen, Ruben S. G. M. Witlox, Arjan B. te Pas

**Affiliations:** ^1^Division of Neonatology, Department of Pediatrics, Leiden University Medical Center, Leiden, Netherlands; ^2^Ritchie Centre, Hudson Institute of Medical Research, Melbourne, VIC, Australia; ^3^Department of Obstetrics and Gynaecology, Monash University, Melbourne, VIC, Australia; ^4^School of Physics and Astronomy, Monash University, Melbourne, VIC, Australia

**Keywords:** resuscitation, neonate, respiratory function monitor, pulse oximetry, video, ECG

## Abstract

Neonatal resuscitation is one of the most frequently performed procedures, and it is often successful if the ventilation applied is adequate. Over the last decade, interest in seeking objectivity in evaluating the infant’s condition at birth or the adequacy and effect of the interventions applied has markedly increased. Clinical parameters such as heart rate, color, and chest excursions are difficult to interpret and can be very subjective and subtle. The use of ECG, pulse oximetry, capnography, and respiratory function monitoring can add objectivity to the clinical assessment. These physiological parameters, with or without the combination of video recordings, can not only be used directly to guide care but also be used later for audit and teaching purposes. Further studies are needed to investigate whether this will improve the quality of delivery room management. In this narrative review, we will give an update of the current developments in monitoring neonatal resuscitation.

## Introduction

During neonatal transition, lung aeration is pivotal for changes in respiratory and cardiovascular function required for survival (1, 2). However, of the 130–136 million infants born annually in the world, approximately 3–5% of term infants and 60% of preterm infants fail to aerate their lungs spontaneously and require some form of resuscitation (3, 4). In most cases, neonatal “resuscitation” comprises only the establishment of adequate ventilation (5, 6), which reflects the importance and vulnerability of infants as they transition to pulmonary gas exchange. Both experimental and clinical studies have demonstrated that even short periods of inappropriate respiratory support can have severe consequences, leading to lung and brain injury, especially in preterm infants (7–9). This injury, which takes place in the first minutes of life, can have a major impact on some of the important morbidities associated with prematurity, such as bronchopulmonary dysplasia and intraventricular/periventricular hemorrhage (10, 11).

The effect of respiratory support should be evaluated and closely monitored in order to stay within the known safe ranges (12). Traditionally, the infant’s condition and effectiveness of resuscitation was evaluated by judging color, chest excursions, and by palpation or auscultation of the infant’s heart rate (HR) (13). But evaluating color, manually counting heart beats, and visualizing chest excursions are inherently inaccurate (14–16), leading to the recommendation that pulse oximetry (PO) or ECG should be used (3, 17). Although experts recommend the use of respiratory function monitoring (RFM) (18), this is not mandated by the international resuscitation guidelines, and in most neonatal units, ventilation is still evaluated by chest excursions (19). However, real-time quantitative information could improve mask technique and ventilation (12).

While regular auditing has been employed to improve quality of the interventions performed by caregivers, this is difficult in delivery room management as few objective parameters can be used and these are usually not recorded. The resuscitation chart is often documented retrospectively, and eyewitness testimonies of stressful events are often inaccurate (20, 21). As a result, high quality documentation can be difficult to obtain and preprinted resuscitation pages in the medical records usually ask for few details. Recording of physiological parameters and video images during neonatal resuscitation could improve quality as auditing is possible and assists in documentation, as it provides detailed information on resuscitation.

In this review, we will describe the current knowledge in monitoring neonatal resuscitation for direct feedback to guide the resuscitator with ongoing care and for feedback later, when it is used for auditing.

## Why Monitor?

Lung aeration is the key factor that initiates pulmonary and hemodynamic changes at birth, which emphasizes the significance of adequate respiratory support/ventilation as the cornerstone of resuscitation/stabilization when transition fails. Management of infant care in the first minutes after birth can severely impact morbidities associated with prematurity, resulting in major adverse outcomes (7–9). Although most clinical trials in delivery room management have failed to show a decrease in morbidity and mortality, there is much experimental data demonstrating that respiratory support given at birth can injure the preterm infant, with potentially life long consequences (7). In addition, large clinical studies in very preterm infants reported higher mortality rates and an increased risk of lung and brain injury with increasing levels of delivery room resuscitation (22–24).

At birth, the lungs of very preterm infants are uniquely susceptible to injury because they are structurally immature, surfactant-deficient, liquid-filled, and not supported by a stiff chest wall (25). Gas volume and lung compliance of newborns greatly changes over the first few breaths as airway liquid is replaced with air. This highlights the large differences in regional lung mechanics that occur when the lung only partially aerates (26). Spontaneously breathing infants develop high transthoracic pressures over the first few breaths, and relatively high positive pressures are required to initiate ventilation (27). However, compliance rapidly increases as more of the lung aerates resulting in lower pressure requirements to achieve a functional tidal volume (V_T_) with subsequent breaths. In addition, although the lung, heart, and brain are often considered independently, they are intimately linked, particularly at birth. Treatments aimed to provide respiratory support can have severe adverse consequences for the preterm heart and brain (7). For example, positive pressure ventilation (PPV) can not only cause lung injury but can also adversely affect the systemic circulation and cerebral circulation. Similarly, inflammation resulting from lung injury can induce a systemic inflammatory response that includes the brain (7).

Hyperoxia can lead to oxidative stress and tissue injury (28, 29). Excessive oxygen exposure should be avoided during stabilization at birth. Recently, updated international resuscitation guidelines recommend that respiratory support in term infants should start with air (29–31). Less clinical data are available for preterm infants, although even a short period of hyperoxia in preterm infants at birth increases oxidative stress and inflammation, and preterm infants initially exposed to a high FiO_2_ have an increased risk for bronchopulmonary dysplasia (32). These findings suggest that in the first minutes after birth, avoiding the use of high oxygen concentrations may reduce acute lung injury, particularly in very preterm infants.

On the other hand, hypoxia is known to inhibit breathing in the fetus due to a direct inhibitory input on the respiratory center of the brain (33). Although a temporal change in O_2_ sensitivity occurs in days/weeks after birth (34) and most preterm infants breathe at birth (35, 36), it is not known when the switch from respiratory suppression to stimulation occurs in response to hypoxia. It is possible that hypoxia immediately after birth will cause a weakened or absent respiratory drive, particularly in preterm infants, who are essentially exteriorized fetuses. Indeed, maturation of the hypoxic sensitivity for breathing is delayed in preterm lambs (34).

In addition, the success of ventilation strategies to support respiratory function at birth in the newborn is largely dependent on the technique and strategy applied. The initial approach is to use non-invasive ventilation applied using a facemask, but most caregivers are not aware or adequately informed concerning effectivity of their mask technique (36).

For the aforementioned reasons, it seems obvious that close monitoring of physiological parameters is necessary to provide adequate respiratory support and keep monitored values in the defined “safe” ranges.

## Direct Feedback

### Pulse Oximetry

When measured by auscultation or by palpation of the umbilical cord, HR is commonly underestimated at birth, possibly because insufficient time is taken to accurately count beats (37). Indeed, studies have observed that caregivers spend only on average 6 s to count (38, 39), which is probably due to the time limits mandated by resuscitation guidelines (19). PO has been recommended for monitoring and evaluating the infant’s condition, and the effect of interventions on infants at birth has replaced assessment of skin color and auscultating HR for evaluation (3). Different PO devices are available; however, no significant differences in HR measurements have been found (40).

Nomograms based on PO measurements have been developed to guide the caregiver during neonatal resuscitation providing acceptable reference ranges (41, 42). PO measurements obtained for nomograms are derived from infants where the umbilical cord was clamped immediately (41). Delayed cord clamping and milking (43, 44) leads to higher SpO_2_ values and lower HR, especially in the first minutes after birth. As delayed cord clamping is now recommended for term infants (19) and implemented in most hospitals worldwide, caregivers should reconsider which nomograms to use. When using the international accepted nomograms, caution should be taken in what we define “healthy” normal transition and which values we find acceptable.

Another concern is that for development of PO nomograms, only good quality data points were used (37, 41). Low quality data [low signal identification and quality (SIQ < 0.3)] and data with low perfusion indices were excluded retrospectively, which comprised 50% of collected data (37, 41). It is obvious that this does not reflect the real situation when clinicians need to evaluate the infant directly after birth and must deal with both high and low quality data. SpO_2_ measurements with low signal quality proved to be reliable for monitoring the infant’s condition, but HR could be underestimated (45).

### ECG

ECG is considered as gold standard for measuring HR, and recent guidelines recommend ECG use, when available, to monitor HR at birth (19). Studies have shown feasibility of collecting ECG data at birth and a reliable signal were obtained faster when compared to PO (17, 46). While HR measurements collected simultaneously by PO and ECG have led to the conclusion that HR measured by PO is accurate (37), a temporal analysis of similar data has shown that PO underestimates HR in the first minutes after birth (17). Difference in HR measured by PO and ECG are mostly likely due to large hemodynamic changes that occur during transition. In the first minutes after birth, especially after immediate cord clamping, cardiac output will be decreased temporarily, which could decrease systolic blood flow pulses that are not detected by PO (17, 47). It is also possible that the direct reversal of the ductal shunt, which occurs directly after birth, diminishes pulse waves peripherally (17, 47). We recommend the use of ECG, when in doubt and when available. However, sensor application can be hampered by body fluids on the skin and care must be taken not to damage the skin. Accuracy of Doppler devices is promising compared to ECG (48). In the future, these devices could be used to determine nomograms instead of relying on nomograms drafted with PO.

### Near Infrared Spectroscopy

The brain is the most important organ of the human body. Monitoring of the neonatal brain could provide additional information during immediate transition and may help to guide resuscitation. Near infrared spectroscopy (NIRS) is the most promising method, since it provides continuous data monitoring and is feasible even in VLBW infants receiving resuscitation interventions (49). Reference values are available for term and preterm infants during transition (50, 51).

### Respiratory Function Monitoring

The use of RFM during resuscitation at birth is currently not recommended in the international guidelines, as there is no evidence that it improves outcome (19). Despite a few trials that are currently running, RFMs are not routinely used in the delivery room to assess adequate neonatal transition and ventilation. Nevertheless, we believe that there are several reasons why the use of a RFM at birth could be helpful during neonatal resuscitation by removing subjectivity involved in evaluating resuscitation (16). Spontaneous breathing is often missed or misjudged, and for this reason, unnecessary ventilation can be given (36, 38). Studies have shown that inflation pressures during ventilation at birth are a poor proxy for actual volumes delivered (36, 52). During transition at birth, pulmonary dynamics change quickly, and with a similar inflation pressure, very different V_T_s can be achieved initially compared with later, during subsequent ventilation. Often inadequate and injuriously high V_T_s occurred during resuscitation (18, 36). Also mask ventilation is often hampered by large mask leak and obstruction (36, 52–56), which was often not noticed by caregivers (36, 52–56).

In a small feasibility study, preterm infants were mask ventilated at birth and randomized as to whether a RFM was visible or not. In the RFM visible group, the mask was more frequently repositioned, and there was a significant decrease in mask leak and intubation as compared to the group where the RFM was not visible (57). Alternatively, combining all physiological parameters [V_T_s, pressures, fractional oxygen given (FiO_2_)] with SpO_2_ and HR onto one screen could increase the chance that caregivers observe the added RFM parameters.

When defining the target range of V_T_s for infants at birth, the following considerations have to be taken into account. The ratio between functional residual capacity (FRC), which is 11 mL/kg, and total lung capacity (19 mL/kg) is smaller in preterm than in term infants (58, 59). Experimental studies have shown that V_T_s > 8 mL/kg distend the lung above total lung capacity and cause lung injury (60, 61). Only five rapid inflations with large V_T_s at birth can cause lung injury (62) and abolish benefits conferred with surfactant treatment (63). Although it is known that over-inflation causes lung injury (64), high inflation rates causing large shear stress are also injurious (65). Many infants can receive inappropriate V_TS_ within minutes of birth (12). Indeed, V_T_s ranged from 0 to >30 mL/kg during resuscitation with a T-piece, with the majority (85%) of preterm infants receiving excessively high V_T_s (>8 mL/kg) (66).

**Figure 1:**
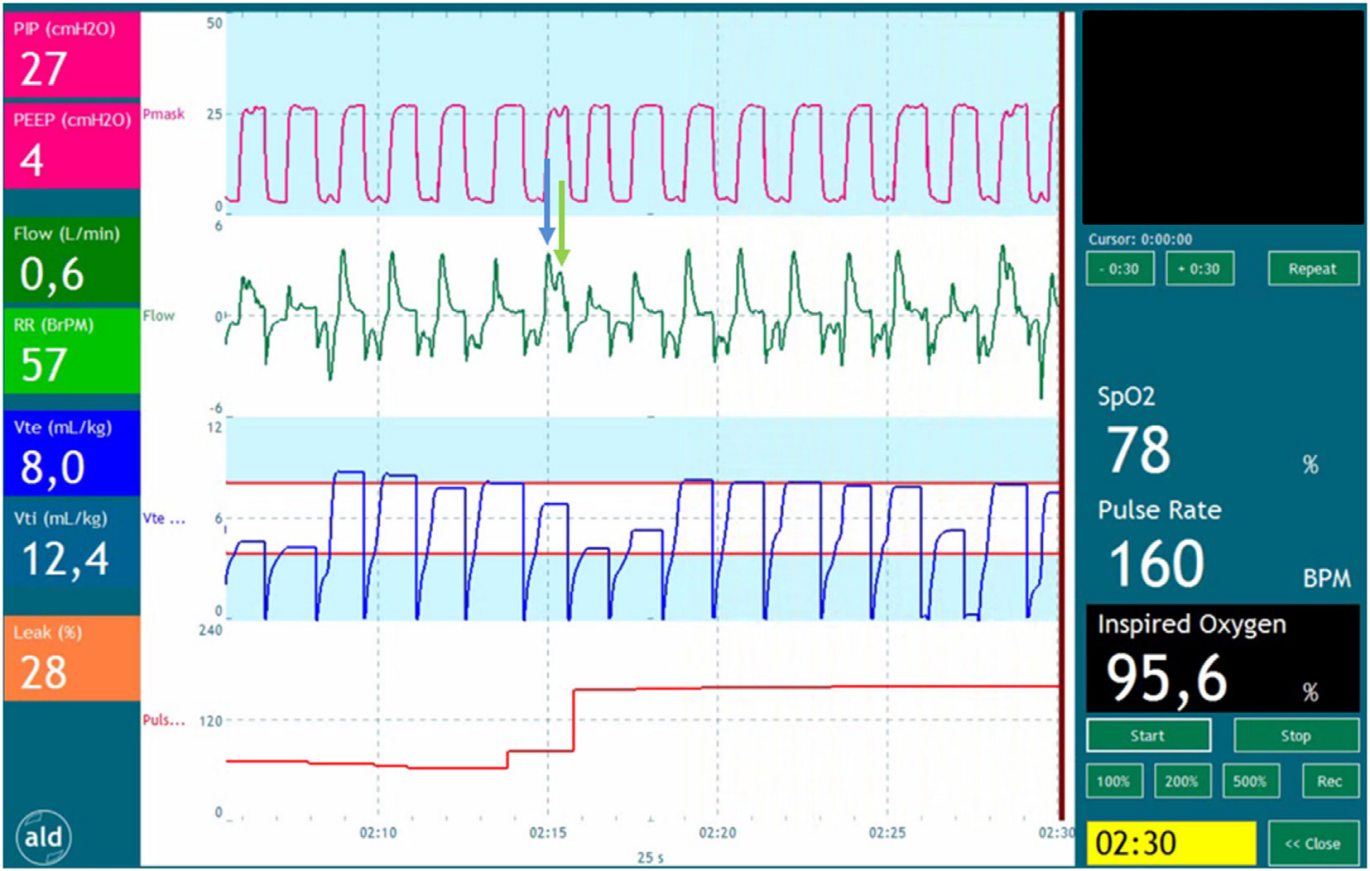
**Recording of a preterm infant receiving positive pressure ventilation**. The tracing shows pressure (red), flow (green), expiratory volume (blue), and pulse rate (red). The blue arrow points a part of the volume entering the oropharynx at the start of the inflation. The green arrow points at the part of volume entering the lungs when the pressure time integral is large enough to overcome the resistance of the glottis and upper airway.

During mask ventilation, the complete respiratory tract (nasopharynx, trachea, and lungs) is pressurized and ventilated. During inflations, there is volume displacement of the nasopharynx, which does not occur during spontaneous breathing (67). This could even lead to V_T_ measurements during ventilation against a closed larynx (Figure 1). During fetal development, laryngeal adduction maintains a higher intra-pulmonary pressure, essential for lung development (68, 69). It is likely that the larynx remains mostly closed at birth, only opened briefly during a breath, which prevents face mask ventilation from inflating the lung (2, 70, 71).

Several studies have shown that spontaneously breathing preterm infants supported only by CPAP, have a mean V_T_ of 4.4 mL/kg (36, 52, 72). Until more data are provided concerning the “safe range,” it seems prudent to use 4–8 mL/kg (12). However, it is likely larger V_T_s are initially needed when lung liquid clearance, lung aeration, and FRC needs to be established compared to the subsequent ventilation when the lungs are aerated and FRC has to be maintained.

### Exhaled CO_2_

Exhaled CO_2_ can be added to monitor lung function at birth. Experimental studies and clinical observations in intubated infants have shown that end-tidal CO_2_ measurements correlated well with V_T_s delivered and amount of lung recruitment (73). End-tidal CO_2_ measurements are taken during the “plateau phase” of each expiration, and this can be influenced greatly when this plateau phase is not reached due to leak. Volumetric measurements, which integrate CO_2_ signal, for the whole inspiration are much more reliable (NM3, Respironics, Eindhoven, the Netherlands).

**Figure 2:**
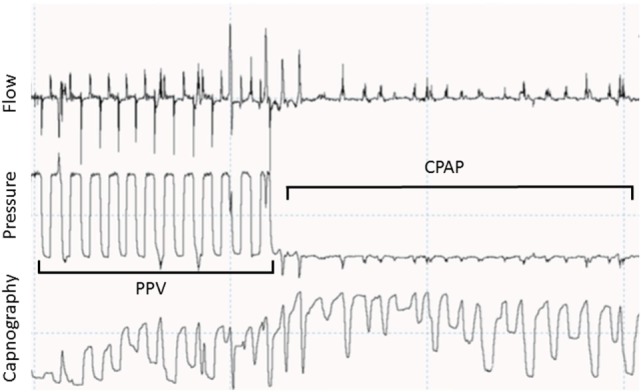
**Recording showing a respiratory tracing showing flow, pressure, and capnography in waveforms**. During positive pressure ventilation (PPV), CO_2_ goes back to 0 during most of the inflations. During spontaneous breathing in continuous positive airway pressure (CPAP) CO_2_ does not go back to 0 due to small tidal volumes and stasis in the sensor.

It was recently showed that spontaneous breathing, added dead space (mask, flow and CO_2_ sensors), mask leak, and laryngeal obstruction during mask ventilation greatly influences exhaled CO_2_ measurements and can easily misinform the caregiver (Figure 2) (74).

Ideally exhaled CO_2_ therefore should be combined with tidal volume measurements to identify adequate ventilation, provided that added dead space is acceptable.

## Late Feedback

Auditing clinical interventions is necessary for quality assurance and should be an integral part of the daily health care. Neonatal resuscitation is commonly audited by using the resuscitation charts documented by the caregiver.

Videotaping emergency medical procedures first emerged in the 1960s (75) and was pioneered in neonatal resuscitation by Finer (76, 77). Videotaping of DR management has shown that 54% of caregivers deviated from the guidelines, most frequently in respiratory management (77). Several items were scored during review of videos, and several deficiencies were frequently detected such as improper detection of chest excursions, suction technique, bag and mask technique, and oxygen use. However, although chest excursions were evaluated by a different caregiver on the video, this remains a subjective measurement for applying adequate ventilation. Regular audits of neonatal resuscitation have been implemented in our unit (Leiden University Medical Centre, the Netherlands), and recording of physiological parameters (SpO_2_, HR, FiO_2_, air flow, pressures, volume and mask leak) were added to the video. During these audits, we observed that caregivers deviated even more often (80%) from the guidelines (7). These deviations most often occurred in the respiratory management (71%; choice of respiratory support, frequency and duration of initial inflations and pressures given) and heat prevention (37% of occasions) (38). This was in agreement with observations in a low-resource setting (78). An important observation was that evaluation of respiratory support and the MRSOPA (*m*ask *r*eposition, *s*uctioning, open *a*irway, increase *p*ressure and consider *a*lternative airway) was frequently not performed. In addition, resuscitation charts often appeared inaccurate or incorrect compared with the recordings (20). Recordings are reviewed plenary with all staff available, on a weekly bases. During this meeting, the intervention is discussed by an independent reviewer after which everyone is invited to give feedback.

### Ethical and Legal Aspects

Recording physiological data and especially video recording are subject to strict legislation concerning privacy. Data collection for audit, however, is subject to less strict rules, as it is intended to improve health care and is a tool for quality assurance. Therefore, it is an integral part of health-care delivery (79). In the United States, the use of video’s without asking written consent is protected by law (80). In Australia and the Netherlands, protocols were assessed by the medical ethical committee, but parental consent was deemed unnecessary (36, 77, 81, 82).

Recording the performance of caregivers during resuscitation can cause concern among staff members. The events of resuscitation could be subject of a disciplinary investigation. This can raise questions with clinicians concerning their anonymity. But de-identification of data could prevent the use for data for legal purposes. Parents very infrequently refuse the use of recordings for audit as well as for research purposes (82). Also, in our experience parents find it acceptable that recordings are made during resuscitation.

### Which RFM to Use?

Although mostly used for research there are different types of RFMs available. The three commercially available monitors use different techniques to measure gas flow and software for calculations of the tidal volume. The most commonly used device in the delivery room is the Florian Neonatal RFM (Acutronic Medical Systems AG, Hirzel, Switzerland) using a hotwire anemometer (11, 13–15), but studies have also been performed with a Respironics Novametrix Non-Invasive Cardiac Output (NICO) monitor (Novametrix Medical Systems Inc., Wallingford, Connecticut), using a differential pressure pneumotachometers with a fixed orifice (11, 12). In the Monitor trial (NTR 4104), a newly developed resuscitation monitor is used with a built-in New Life Box Neo-RSD (NLB Neo-RSD, Advanced Life Diagnostics UG, Weener, Germany) for lung function measurements, using a differential pressure pneumotachometers with a fixed orifice. Also a low cost monitor has been developed (Laerdal Newborn Resuscitation Monitor, Laerdal, Stavanger, Norway) using a hot wire anemometer and is recently used in studies in a low-resource setting (83).

## Conclusion

Although perinatal caregivers are trained for neonatal resuscitation, it remains a stressful event. Inadvertently, potential injurious interventions can be given by the caregiver during the infant’s most vulnerable moment when it is undergoing a major physiological transformation, especially in preterm infants. Traditional parameters on which the infant’s condition and the effectiveness of interventions are assessed have shown to be subjective and inaccurate. Monitoring physiological parameters is feasible, easy to do, and gives an objective feedback directly during resuscitation or later when it is used for audit. This feedback will improve performance of caregivers during neonatal resuscitation, but further studies are needed to determine whether this will improve patient outcomes.

## Author Contributions

JV and AP wrote the first draft of the manuscript, critically reviewed the manuscript, and approved the final version. HZ, KS, SH, MK, and RW critically reviewed the manuscript and approved the final version.

## Conflict of Interest Statement

The authors declare that the research was conducted in the absence of any commercial or financial relationships that could be construed as a potential conflict of interest.
